# Pregnancy in Women with Atopic Dermatitis: A Systematic Review of Concerns and Challenges

**DOI:** 10.2340/actadv.v106.adv-2026-0608

**Published:** 2026-05-18

**Authors:** Line B Nørreslet, Tove Agner, Charlotte G Mortz

**Affiliations:** 1 Department of Dermatology and Allergy Centre, Odense University Hospital, University of Southern Denmark, Odense, Denmark; 2 Department of Dermatology, Bispebjerg Hospital, University Hospital of Copenhagen, Copenhagen NV, Denmark

**Keywords:** atopic dermatitis, atopic eczema, atopy, pregnancy, family planning

## Abstract

Atopic dermatitis has negative individual and societal implications. However, the potential impact of atopic dermatitis on reproduction has only been sporadically investigated. We aimed to provide an overview of pregnancy in atopic dermatitis, with a specific focus on concerns, challenges, the number of children and adverse pregnancy and birth outcomes. A systematic literature search was performed using EMBASE and PubMed up until 19 January 2026 for original articles in relation to reproduction in female atopic dermatitis. Twenty-four highly heterogeneous studies were included. Concerns about atopic dermatitis in relation to pregnancy and family planning were reported in >50% of the patients in 3 out of 4 studies. Conflicting results were seen regarding fertility and disease severity during pregnancy. Pregnancy rates were similar or increased based on 2 studies. Eleven studies addressed adverse pregnancy and birth outcomes, of which 5 studies unanimously reported a protective or indifferent effect of atopic dermatitis on pre-term birth, gestational diabetes and stillbirth. Outcomes such as gestational hypertension, preeclampsia and miscarriage showed divergent results. Overall, atopic dermatitis causes considerable worries in relation to family planning. However, this review implies that pregnancy rates as well as complications in pregnancy and during childbirth in women with atopic dermatitis overall are consistent with women without atopic dermatitis.

**SIGNIFICANCE**

Atopic dermatitis is the most prevalent chronic skin disease in pregnant women. The association between atopic dermatitis in pregnant women and reproductive outcomes remains unclear due to conflicting research findings. The review summarizes that patients with atopic dermatitis have high levels of concern in relation to pregnancy and reproduction. However, atopic dermatitis is not consistently linked with reduced fertility or adverse pregnancy and birth outcomes. Therefore, this review implies that women with atopic dermatitis should be informed and reassured with respect to disease progress as well as pregnancy complications and outcome before and during pregnancy.

Atopic dermatitis (AD) is a relapsing, inflammatory skin disease with a prevalence of 20% in children and 2–10% in adults in developed countries (1). In adults, the prevalence of AD peaks around the childbearing age of 25–44 years, with females being overrepresented (1, 2). The burden of AD includes considerable socio-economic and personal costs with negative impact on the healthcare system (3), health-related quality of life, including sexual health (4, 5), educational achievement (6) and work life (7, 8). Relatively few studies have assessed adult women with AD before and during pregnancy in relation to reproduction.

Family planning may cause concerns for AD patients, as previously described for patients with other chronic inflammatory skin diseases (9). Uneasiness may include disease worsening due to treatment limitations, a potential risk to the fetus, and a fear of passing the disease on to their offspring (4, 10, 11). The desire to conceive may be negatively affected, potentially delaying the time to pregnancy, as observed in other atopic diseases (12). Moreover, patients with AD may have fewer children than looked for, either by choice or due to biologic infertility (13). Maternal and fetal complications during pregnancy and birth have been suggested to occur (5), although not confirmed. Additional knowledge in this field is important to improve the management and guidance of women with AD regarding the expected course of pregnancy. Likewise, identifying knowledge gaps in this field is fundamental for future research.

The aim of this review was to systematically examine the current literature regarding the impact of AD on female reproduction, with a specific focus on 1) concerns, 2) challenges, 3) maternal age and the number of children and 4) adverse pregnancy and birth outcomes. The hypotheses were that women with AD are noticeably concerned before and during pregnancy, are older when they conceive, have fewer children, and experience more complications during pregnancy and birth compared to the general population.

## MATERIALS AND METHODS

### Literature search strategy

This study was conducted in accordance with the Preferred Reporting Items for Systematic Reviews and Meta Analyses (PRISMA). The protocol created prior to data extraction was published and is available at PROSPERO (https://www.crd.york.ac.uk/PROSPERO/view/CRD420250651360). The literature search was performed using databases (PubMed and EMBASE) from their inception through 19 January 2026. Additionally, literature was sought from reference lists of included studies and review articles. A broad search strategy using search terms in relation to AD and pregnancy including concerns, disease severity, number of children, age at first childbirth, fertility, miscarriage and complications in pregnancy, during birth and postpartum was performed (Table SI). These search terms and their synonyms served as inclusion criteria. The results were filtered to include articles with humans and English language only. All epidemiological and clinical study designs were eligible for inclusion. Case reports and studies evaluating pregnancy and/or birth outcomes following exposure to treatment of atopic dermatitis were excluded as it was outside the scope of this review.

Two reviewers (LBN and CGM) independently performed selection of eligible articles using Covidence (14) screened by title and/or abstract and then by full-text reading. Disagreements were resolved by discussion.

### Data extraction and quality assessment

Data extraction included items as follows: country, journal, author, publication year, study design, population (age, sex, AD), how AD was diagnosed, severity of disease and the relevant reproductive outcomes and results in relation to AD. A quality assessment was performed for each study included using a fixed validated list of criteria (15) with modifications to also fit cross-sectional studies included in this review (7). Study design, bias and analyses weighted more in the overall classification of study quality. Due to heterogeneous results meta-analysis was not suitable.

## RESULTS

**Fig. 1.**
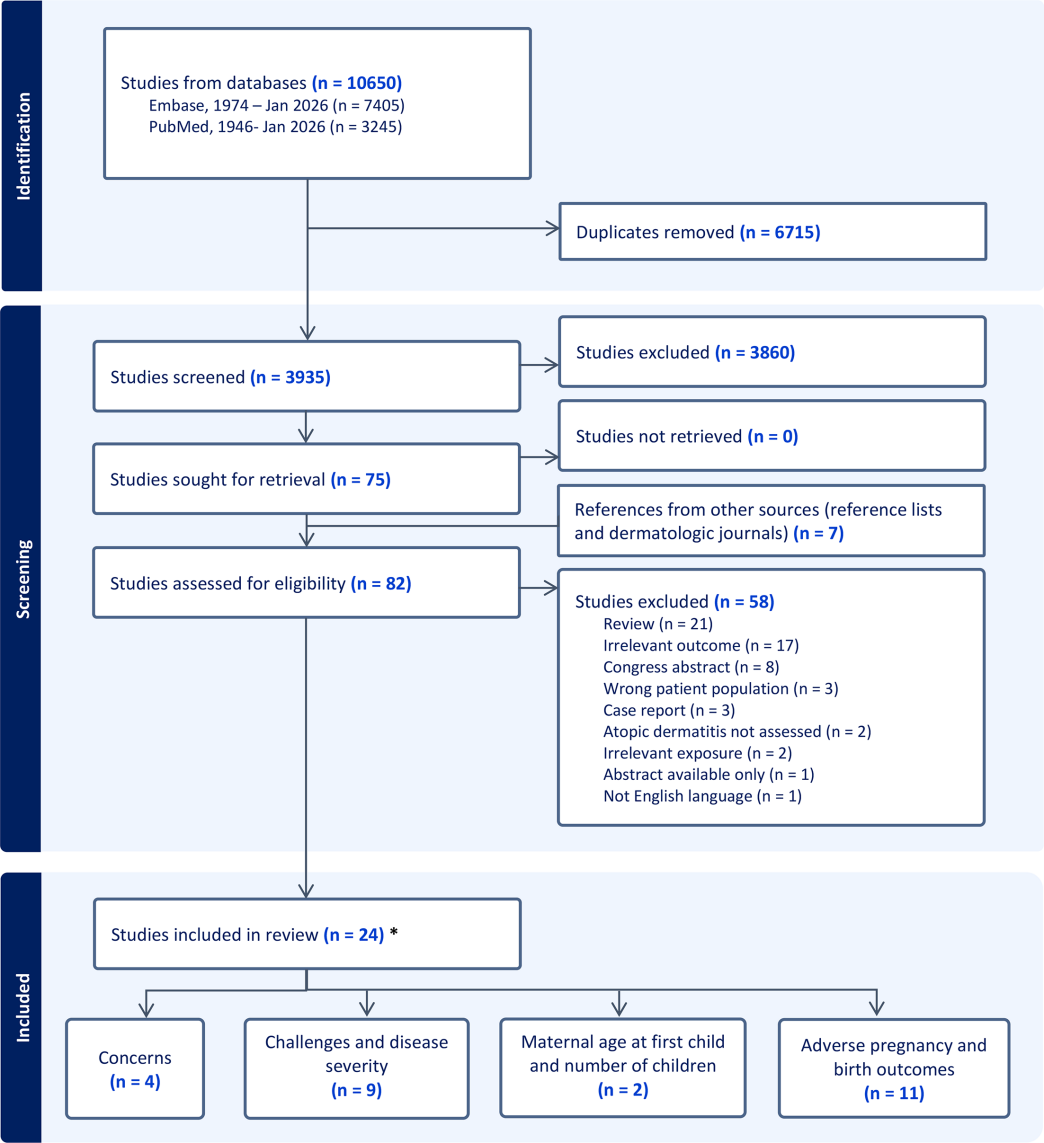
Preferred Reporting Items for Systematic Reviews and Meta-Analysis (PRISMA) diagram. *Two studies (16, 17) included assessed two of the outcomes reviewed.

The literature search yielded 3,935 nonduplicate records for screening. Review of the titles and abstracts excluded 3,860 articles, and following full-text review of 75 articles, additional 58 articles were excluded. The reference lists of the included articles for full-text review were screened, and recent dermatologic journals were screened, resulting in seven additional studies included. In total, 24 studies were eligible for final assessment. Two studies assessed more than one of our four predefined outcome categories (16, 17). Four studies evaluated concerns in relation to reproduction in patients with AD (Table I), 9 studies assessed challenges and disease severity of AD during pregnancy (Table II), 2 studies investigated childlessness (Table II), and 11 studies reported adverse pregnancy and birth outcome of maternal AD (Table III). The process and reasons for the studies excluded are illustrated with a PRISMA flow diagram (Fig. 1).

**Table I.** Studies on concerns in relation to pregnancy for patients with atopic dermatitis. Listed by publication year

Reference Author, year, country (journal)	Study design	Population	AD diagnosis AD severity	Outcomes	Results	Quality assessment
(11) Skovsgaard et al., 2025, Denmark (Acta DV)	Cross-sectional Questionnaire Consecutively recruited	N(AD)= 121, men and women, 18–45 years	Diagnosis: Doctor Severity: EASI (Mean 4.6, 85% in systemic treatment)	Concerns of AD in relation to pregnancy and/or breastfeeding: 1) Any concern 2) Potential heritability 3) Teratogenic side effects of treatment 4) Breastfeeding 5) Fewer children than desired	1) 89% 2) 88% 3) 30% 4) 37% 5) 16%	*Moderate/low* Strengths: Patients consecutively recruited Diagnosis and severity by doctor Limitations: Cross-sectional design No reference group Small sample size limits subgroup analyses
(18) Fuentes-Barragán et al., 2025, Spain (Acta DV)	Cross-sectional Questionnaire Consecutively recruited	N(AD)= 104, men and women, ≥18 years	Diagnosis: Doctor Severity: moderate to severe (mean): EASI (14.3), SCORAD (37), BSA (23), POEM (15), WHO-5 (14.2), DLQI (8.8)	Impact of AD on: 1) the desire to have children 2) on having children 3) on having as many children as desired	1–3) No impact of AD, and no significant difference for mild-moderate vs. severe AD	*Moderate/low *Strengths: Patients consecutively recruited Diagnosis and severity by doctor Limitations: Cross-sectional design No reference group
(4) Fougerousse et al., 2024, France (Acta DV)	Cross-sectional Questionnaire Retrospective	N(AD)= 1,009 women, ≥18 years	Diagnosis: Self-reported doctor-diagnosed Severity: POEM (50.5 % mild, 39.3 % moderate and 10 % severe)	1) Pregnancy concern due to AD, overall 2) AD impact the desire to have a child 3) AD delay pregnancy 4) Fear of passing AD on to the child 5) Breastfeeding concerns related to AD	1) 74 (7.3%) 2) 41/74 (55%) 3) 31/74 (42%) 4) 67/74 (91%) 5) 93.7 % reported that the decision to breastfeed was unrelated to AD	*Moderate/low* Strengths: Large sample size Response rate 82% Limitations: Cross-sectional design No reference group Not validated questionnaire Retrospective and self-reported data (selection and recall bias)
(24) Rodriguez-Pozo et al., 2024, Spain (ActaDV)	Cross-sectional Questionnaire Retrospective	N(AD)= 102 women, ≥18 years	Diagnosis: Self-reported Severity: Patient-Oriented SCORAD (mean 55.52, (i.e.) severe), DLQI (mean 18.5), POEM (mean 16), WHOQOL (mean 43)	The impact of AD on reproductive wishes in women: 1) Gestational desire 2) Factors associated with impairment in gestational desire	1) 51 % reported influence 2) Associated with: - Gluteal involvement of AD - Civil status single (vs. married) - Worse DLQI	*Low* Strengths: Severity assessed Statistical tests and data analyses specified and relevant Limitations: Cross-sectional design No reference group Sample size relatively small Selection bias Patient-reported outcomes

AD: atopic dermatitis; BSA: Body Surface Area; DLQI: Dermatology Life Quality Index; EASI: Eczema Area and Severity Index; n.a.: not assessed; n.s.: not significant; POEM: Patient-Oriented Eczema Measure; SCORAD: SCORing Atopic Dermatitis; WHO-5: World Health Organization-Five Well-Being Index; WHOQOL: World Health Organization Quality of Life.

**Table II.** Studies on challenges and disease severity in relation to pregnancy as well as fertility rates for patients with atopic dermatitis. Listed by publication year

Reference Author, year, country (journal)	Study design	Population	AD diagnosis AD severity	Outcomes	Result	Quality assessment
(28)^*^ Kjersgaard et al., 2025, Denmark (Human Reproduction Open)	Cohort Questionnaire, i.e., self-reported outcomes	*N*= 88,713 pregnant women; 3,364 (4.1%) AD	Diagnosis: self-reported, doctor-diagnosed Severity: n.a.	AD vs non-AD: 1) Time to pregnancy>12 month 2) conceiving using infertility treatment	1) RR ratio 0.83 (95%CI: 0.72–0.96) 2) RR ratio 0.81 (95%CI: 0.77–0.94)	Moderate/high Strengths: National, large population Prospectively collected Adjustments for potential confounders Information on active AD in pregnancy Limitations: Self-reported data, risk of recall bias Categorical outcomes No information on treatment or severity
(17)^*^ Schmidt et al., 2025 Denmark (JEADV)	Registry Cohort	N(AD)= 8,409 N(non-AD)=853,228 men and women, ≥30 years	Diagnose: Hospital-diagnosed Severity: Systemic treatment or phototherapy as severe	AD vs non-AD: 1) Childlessness by age 30 2) Assisted reproduction by age 30	1) 57.7 % vs 56.7%, adj OR 1.01 (95%CI: 1.00–1.03) 2) 3.8 % vs 3.3%, adj OR 1.01 (95%CI: 0.89–1.15)	*Moderate/high* Strengths: National, large-scale, registry Adjustments for covariates Sibling cohort to control for family-related confounding Limitations: Potential misclassification (mild AD as control)
(20)^*^ Liu et al., 2023, Finland and Sweden (Nat Hum Behav)	Registries from Finland and Sweden Cohorts	N(AD)= 36.132 N(non-AD)=2,545,020 men and women, ≥16 years	Diagnose: Hospital-diagnosed Severity: n.a.	Childlessness (biologic children) in AD vs non-AD: 1) At age 45 for women 2) at age 50 for men	1) OR 0.95 (95%CI: 0.83–1.09) 2) OR 1.05 (95%CI: 0.91–1.21)	*Moderate/high* Strengths: Two national, large-scale, registries Adjustments for covariates Sibling cohort to control for family-related confounding Limitations: Potential misclassification (mild AD as control) No distinction between voluntary or involuntary childlessness
(29) Rakita et al., 2022, USA (JAAD)	Cross-sectional Questionnaire (worsening of AD during pregnancy, retrospectively) Prospectively recruited	N(AD)= 211 women, ≥18 years; 68 (32%) with a previous pregnancy	Disease: Dermatologist-diagnosed Hanifin-Rajka criteria Severity at recruitment: EASI, IGA, BSA	Worsening of AD during pregnancy	12 (17.7%) patients	*Moderate/low* Strengths: Doctor-evaluated diagnosis and severity Limitations: No reference group Sample size small Recall bias
(19) Horev et al. 2021, Israel (Dermatology)	Registry Cohort with matched controls	N(AD)= 127,150 men and women, of which 42,548≥18 years N(non-AD)=127,071	Diagnosis: Dermatologist Severity: Based on medication and healthcare utilization (7.1 % severe)	AD vs matched non-AD with respect to infertility: 1) All ages, both sexes 2) Adult AD 3) Adult moderate-to-severe AD	1) 1.4 % vs 1.1%, *p*<0.05 2) 4.2%, *p*<0.05 3) 3.9%, *p*>0.05	*Moderate* Strengths: Cohort with matched controls Large sample size Severity assessment Limitations: Retrospecitve Missing data: The database covers half of Israel’s population; No information on patient’s partnership status or desire to conceive
(27) Olsson Magi et al., 2019, Sweden (ERJ Open)	Prospective birth cohort (pregnant women enrolled at week 18) Questionnaire (mid and late pregnancy)	*N*= 2,164 pregnant women from general population; 426 (20%) AD	Diagnosis: Self-reported doctor-diagnosed Severity: N.a.	AD associated with maternal stress in mid and late pregnancy	High stress score in late pregnancy was associated with symptoms of AD	*Moderate* Strengths: Prospective No recall bias Validated measurement for stress in pregnant women Adjusted for confounding factors Limitations: Disease severity n.a. Small number of subjects with AD symptoms (*n*=30) weakening the results of interest in this review
(25)^*^ Cho et al., 2010, Korea (Ann Dermatol)	Cross-sectional Retrospective Questionnaire Recruited from outpatient clinic database	N(AD)= 97 women, ≥20 years; 23 patients with≥1 pregnancy	Diagnosis: Hanifin and Rajka criteria Severity: EASI (mean 12.3)	Deterioration/ improvement of AD during pregnancy	14 (61%) deterioration 1 (4%) improvement 8 (35%) no change	*Low* Strengths: Severity assessed Limitations: Retrospective design, recall bias No reference group Small sample size (23 pregnant)
(30)^*^ Tata et al., 2007, United Kingdom (Am J Epidemiol)	Registry Cohort	*N*= 491,516 women, 15–44 years; 68,764 (14%) AD	Diagnosis: Doctor-diagnosed in medical records Severity: n.a.	Fertility rates in women with AD vs non-AD	59.4 vs 51.0 livebirths per 1,000 person-years Fertility rate ratio: 1.15 (95%CI: 1.13–1.17)	*Moderate/high* Strengths: National, large-scale, registry Adjustments for covariates Limitations: Retrospective (associations, not causative conclusions) Disease severity, duration and treatments n.a.
(26) Kemmet and Tidman, 1991, United Kingdom (Br J Dermatol)	Cross-sectional Retrospective Questionnaire	N(AD)= 156 women, 15–45 years; 50 (33%) patients with≥1 pregnancy	Diagnosis: Hanifin and Rajka criteria for AD Severity: n.a.	Disease severity of AD during pregnancy	52 % (*n*=26) deterioration, the majority before week 30 24 % (*n*=12) no change 24 % (*n*=12) improvement	*Low* Strengths: Moderate/high response rate Limitations: 43 years old study Retrospective design Disease severity n.a. No controls/references

*From reference lists or screened in recent dermatological journals.

AD:atopic dermatitis; adjOR:adjusted OR; BSA:Body Surface Area; CI:confidence interval; EASI:Eczema Area and Severity Index; n.a.:not assessed; n.s.:not significant; OR:odds ratio; RR:relative risk; vs:versus.

**Table III.** Studies on adverse pregnancy and birth outcomes in patients with atopic dermatitis. Listed by publication year

ReferenceAuthor, year, country(journal)	Study design	Population	AD diagnosis AD severity	Outcomes	Result	Quality assessment
(35) Isogami et al., 2024, Japan (Maternal and Child Health Journal)	Prospective birth cohort study Questionnaire and medical records	*N*=87,796 women with singleton pregnancies; 13,261 AD	Diagnosis: Self-reported questionnaire Severity: n.a.	AD association with preterm birth before week: 1) 37 2) 32 3) 28	OR 0.89 (95%CI: 0.81–0.98) (i.e.) decreased incidence of preterm birth 1) OR 0.98 (95%CI: 0.74–1.30) 2) OR 0.88 (95%CI: 0.50–1.55)	*Moderate* Strengths: Prospective cohort Large sample size Adjusted for several, relevant confounding factors (maternal demographics and socioeconomic status) Limitations: Misclassification bias (diagnosis self-reported) and potentially overestimation bias Severity n.a. Women with multiple abortions, stillbirths were excluded No information on preterm birth risk factors
(31) Weng et al., 2024, Taiwan (Journal der Deutschen Dermatol. Gesellschaft)	Registry Cohort Maternal and offspring cohorts	*N*(AD)=15,495 pregnant women, 15–50 years N(non-AD)=77,475	Diagnosis: Registry Severity: N.a.	AD association with: 1) Threatened abortion 2) Gestational diabetes mellitus 3) Gestational hypertension 4) Preeclampsia/ eclampsia	1) aHR 1.14 (95%CI: 1.10–1.18) 2) aHR 1.06 (95%CI: 0.98–1.15) 3) aHR 1.09 (95%CI: 0.92–1.27) 4) aHR 1.13 (95%CI: 1.01–1.27)	*Moderate* Strengths: National, large-scale, registry Information on several covariates Limitations: Retrospective (associations, not causative conclusions) Misclassification (diagnostic inaccuracies) Disease severity and duration n.a.
(32) Barbieri et al., 2022, (Letter) USA (JID Innovations)	Retrospective cohorts Two different databases (Optum and THIN)	Women, 15–45 years Preeclampsia analysis, Optum cohort: N(AD)=1,821 N(non-AD)=7,946 THIN cohort: N(AD)=142,773 N(non-AD)=515,542	Diagnosis: Registry Severity: n.a.	AD vs non-AD: Preeclampsia	Optum cohort: 1.59% vs 1.10%, OR 1.27 (95%CI: 0.81–1.94); THIN cohort: 0.74% vs. 0.67%, OR 1.11 (95%CI: 1.03–1.19) Meta-analysis: OR 1.11 (95%CI: 1.03–1.19)	*Moderate* Strengths: Two independent cohorts with matched reference groups Large sample sizes Limitations: Retrospective Limited adjustment for confounding factors
(34) Kojima et al. 2021, Japan (Pediatrics International)	Prospective birth cohort Questionnaire	*N*=81,791 pregnant women; 12,951 (15.8%) AD	Diagnosis: Self-reported clinician-diagnosed Severity: N.a.	AD association with: 1) Threatened abortion 2) Preterm birth at a) 34–36 weeks or b) 22–33 weeks	1) adjOR 1.03 (95%CI: 0.97–1.10) 2 a) adjOR 0.89 (95%CI: 0.79–1.01) 2b) adjOR 1.01 (95%CI: 0.80–1.27) No significant associations	*Moderate* Strengths: Prospective design Large sample size Statistical analyses with adj. for numerous confounders Limitations: Self-reported lifetime prevalence, i.e., not necessarily active disease while pregnant Severity n.a. Treatments unknown
(36) Saito-Abe et al., 2021, Japan (Int Arch Allergy Immunol)	Cross-sectional Questionnaire to a birth cohort Outcomes identified by obstetrician (medical records) National multicentre	*N*=97,683 pregnant women consecutively recruited in first trimester; 15,251 (15.7%) AD	Diagnosis: Self-reported Severity: N.a.	AD association with: 1) Preterm birth (at week 34–36) 2) Threatened preterm labour 3) Preterm premature rupture of the membrane	1) OR=0.86 (95%CI: 0.78–0.95) 2) No association 3) No association	*Moderate/high* Strengths: Large sample size Statistical analyses with adj. for numerous confounders Limitations: Self-reported lifetime prevalence, (i.e.) not necessarily active disease while pregnant/at birth delivery Severity n.a. Treatments unknown
(5) Hamann et al., 2018, Denmark (JEADV)	Registry Retrospective cohort with matched controls	N(AD)=10,441 women,≥18 years N(non-AD)=104,410	Diagnosis: Registry Severity: Based on treatment information	AD vs non-AD: 1) Obstetric outcomes: a) placental abruption b) premature rupture of membranes c) eclampsia 2) birth outcomes: a) stillbirth b) gestational age at birth c) weight at birth d) staphylococcal neonatal septicemia 3) pregnancy outcomes: a) hypertensive diseases b) gestational diabetes	1b) OR 1.15 (95%CI: 1.05–1.27) 1 c) OR 2.25 (95%CI: 1.15–4.42) 2d) OR 2.45 (95%CI: 1.33–4.49) 3 a) OR 1.11 (95%CI: 1.01–1.22) 3b) OR 0.79 (95%CI: 0.68–0.92) Other outcomes: n.s.	*High/moderate* Strengths: Cohorts with matched controls Large sample size Information on treatments and AD activity/severity in relation to pregnancy Statistical analyses adjusted for covariates Limitations: Non-hospital diagnosed AD are not included and could be included among controls No causative conclusions
(23)^*^ Saito et al., 2018, (Letter) Korea, Japan (Allergy)	Prospective birth cohort National Multicenter	*N*=90,206 women/newborns with full-term singleton birth; 14.252 (15.8%) maternal AD	Diagnosis: Self-reported Severity: N.a.	AD association with small-for-gestational age birth	adj OR 1.11 (95%CI 1.00–1.24), *p*=0.048	*Moderate* Strengths: Prospective Reference group Large sample size Adjusted for confounders Limitations: Disease self-reported and not necessarily in relation to pregnancy Severity n.a.
(22) Trønnes et al., 2014, Norway (Paediatr Perinat Epidemiol.)	Registry All births in Norway 1967–2003	*N*=1,974,226 births; 66,535 (3.4%) maternal AD	Diagnosis: Registry Severity: n.a.	AD association with: 1) Preterm birth (before 37 weeks” gestation) 2) Stillbirth 3) Neonatal death 4) Preeclampsia, unspecified bleeding, mean birthweight	1) RR 0.90 (95%CI: 0.86–0.93) 2) RR 0.70 (95%CI: 0.62–0.80) 3) RR 0.76 (95%CI: 0.65–0.90) 4) No significant differences AD vs non-AD	*Moderate/high* Strengths: National, large-scale registry Cohort and reference group Adjusted analyses for potential confounding factors Limitations: Misclassification (maternal AD may be underreported to the birth registry in case of a severe birth event) Disease severity or activity during pregnancy/birth n.a.
(33) Sugiura-Ogasawara et al., 2013, Japan Obstet. Gynaecol. Res.)	Population-based cohort study with Medical examination Questionnaire	*N*=2,733 women, 35–79 years; 1,429 with a history of AD	Diagnosis: Self-reported Severity: n.a.	AD association with: 1) Miscarriage 2) ≥2 consecutive recurrent spontaneous abortion	1) OR 1.44 (95% CI: 1.05–1.99) 2) OR 1.42 (95%CI: 0.69–2.92)	*Moderate/low* Strengths: Population-based Reference group Limitations: Objective/outcome extracted for the purpose of this review, it was a minor result in this study Selection bias/low response rate No information on AD before/after pregnancy Analyses poorly described and not adjusted for allergic rhinitis
(16) Seeger et al., 2006, USA, Spain (Dermatology)	Registry Cohort with controls	N(AD)=3,261 women, 10–49 years N(no skin disease)=43,861	Diagnosis: Registry Severity: Based on treatment information	AD vs no-skin-disease: 1) Pregnancy-rates 2) Spontaneous abortion (miscarriage) 3) Therapeutic abortion 4) Stillbirth	Mild AD 5.1 (95%CI: 4.4–5.8) pregnancies per 100 women year, Severe AD 4.5 (95%CI: 3.2–6.2) vs Controls 4.3 (95%CI: 2–4.5) 1) 20.3 % vs 17.2% 2) 6.3 % vs 7.0% 3) 0 % vs 0.6% No significant differences	*Moderate* Strengths: Registry, large-scale study with matched controls Limitations: Selection bias (middle-class insured population included) Treatment allocation based on 6 months and disease severity could be misclassification Mild AD underrepresented Not adjusted for asthma and allergic rhinitis, SES
(21)^*^ Savilahti et al., 2004, Finland (Clin Exp Allergy)	Registry on infants born in three hospitals in Helsinki 1995–1999 Questionnaire to father and mother	N(very low birth weight)=418; 177 with maternal or paternal AD N(birth weight>3000 g)=616; 384 with maternal or paternal AD	Diagnosis: Self-reported doctor-diagnosed Severity: n.a.	AD of mother and father, respectively, and risk of very low birth weight<1500 g	No significant associations	*Moderate/low* Strengths: Moderate sample size Cohort and reference group Limitations: Self-reported diagnosis Severity and presence of AD during pregnancy n.a. Analyses not adjusted for confounders Selection bias (responders to questionnaire)

*From reference lists or screened in recent dermatological journals.

AD:atopic dermatitis; adjOR:adjusted OR; aHR:adjusted hazard ratio; CI:confidence interval; n.a.:not assessed; n.s.:not significant; OR:odds ratio; RR:relative risk; vs:versus.

### Study characteristics

The studies included were heterogenic with respect to objectives, designs and outcome parameters, and no randomized controlled studies were identified. The study designs were retrospective or prospective, cross-sectional, cohorts, and register-based with or without matched control groups. Publication year ranged from 1991 to 2025. Collectively, the 24 studies included 7,107,891 individuals from 12 different countries worldwide, including Scandinavia, Europe, the US, Asia and the Middle East, comprising 459,414 individuals with AD. All study populations included women of childbearing age, and 7 studies additionally included men (11, 17–22). Three studies identified infants from a birth cohort and related the outcomes with maternal AD (21–23), 2 studies included children as well (16, 19). Disease severity was assessed in few studies and by dissimilar definitions including physician’s or patient’s evaluation, or by the treatments used.

### Concerns

The four studies addressing concerns in relation to pregnancy among patients with AD were cross-sectional, questionnaire-based studies without reference group. In 2 studies, patients were recruited consecutively (11, 18), while the 2 other studies were conducted retrospectively (4, 24). The studies were conducted in Europe in 2024–2025, the populations were aged 18 years or above, and descriptive statistics were used. Three studies found that 51–89% of the patients had concerns with respect to pregnancy and family planning in relation to AD (4, 11, 24), while 1 Spanish study of 104 women and men consecutively recruited, did not (18). An overview is presented in Table I.

In the two recent studies from Denmark (*n*=121 women and men) and France (*n*=1,009 women), respectively, the reported concerns were fear of heritability of AD (88–91%) (4, 11) and teratogenic side effects of AD treatment (30%) (11) among the patients with AD. In the French study, 55 % reported that AD had impact on the desire to have children, and 42 % found that concerns related to AD delayed the time to pregnancy (4). In the Danish study, 16% of AD patients had fewer children than desired, largely due to the concerns of hereditary transmission of AD, exacerbation of AD during pregnancy, and the need to discontinue AD treatment (11). On the contrary, the patients in the Spanish study (18) reported no impact of AD on the desire for children, nor for having as many children as desired, and the findings were not related to AD severity. Conflicting results were found regarding concerns of breastfeeding in relation to AD (4, 11).

### Challenges and disease severity

Nine studies considered challenges and disease severity in relation to pregnancy in women with AD (Table II). Four studies reported a negative impact of AD on either fertility (19), disease worsening (25, 26) or maternal stress (27) during pregnancy, while five studies did not (16, 17, 28–30).

An Israeli registry study from 2021 investigated infertility, defined as at least one documented diagnosis of infertility in the medical record, and compared women and men with AD (*n*=42,548) with matched healthy controls (19). The study found a significantly higher prevalence of infertility among the patients with mild-to-severe AD (19). A large Danish questionnaire-based cohort study published in 2025 investigated pregnant women (*n*=88,713) and found that the 3,364 women with AD were associated with a decreased risk of postponing pregnancy, (i.e.) waiting for more than 12 months to conceive (relative risk ratio 0.83, 95% CI: 0.72–0.96) and were significantly more unlikely to receive infertility treatment before conceiving (relative risk ratio 0.81, 95% CI: 0.77–0.94), respectively (28). Another recent Danish registry study compared adults with and without AD and found no difference in assisted reproduction by the age of 30 (17).

Two registry studies, published in 2006–2007, assessed pregnancy rates in women with AD. A Spanish study identified a cohort of females with AD aged 10–49 years (*n*=3,261) and a comparison group (*n*=43,861) to compare the pregnancy-rates (16). No statistically significant differences were found between women with mild AD, severe AD and controls with 5.1 (95%CI: 4.4–5.8), 4.5 (95%CI: 3.2–6.2) and 4.3 (95%CI: 2–4.5) pregnancies per 100 women year, respectively. Another, larger, study from the United Kingdom documented a significantly higher fertility rate in women with versus without AD (fertility rate ratio 1.15, 95% CI: 1.13–1.17) (30).

Three cross-sectional, questionnaire-based studies investigated disease severity of AD during pregnancy (25, 26, 29). Two retrospective studies from 2010 (25) and 1991 (26) including 23 and 50 pregnant women, respectively, reported deterioration of AD during pregnancy in 61% and 52%, respectively, while the 3rd study from 2022 (29), comprising 68 patients reported deterioration of AD during pregnancy in 17.7% only. Few patients reported improvement in AD during pregnancy.

Maternal stress in pregnancy was assessed in a Swedish prospective questionnaire-based cohort study including 2,164 women of which 426 (20%) had AD. High stress score in late pregnancy was significantly associated with AD symptoms, as an indicator for deterioration (27).

### Maternal age at first child and number of children

No studies directly investigated the maternal age at 1st child nor the number of children in women with AD, although one study with self-reported AD used the maternal age to adjust for fecundity analyses (28). They reported maternal age at birth to be 30,0 and 30,5 years for mothers with and without AD, respectively. Two large, Scandinavian registry studies found no association between AD and childlessness at the age of 30 years (17) or the age of 45 years (20), respectively (Table II).

### Adverse pregnancy and birth outcomes

With respect to the influence of AD and adverse events in pregnancy and birth, 11 studies were included, and in this category, objectives and outcomes were highly heterogeneous (Table III). The study designs were diverse, although ten studies were based on registries or birth cohorts with large patient populations, whereas a single study was a population- and questionnaire-based cohort study.

Some frequently occurring complications in pregnancy were assessed. In two studies from Denmark (*n*_AD_=10,441) (5) and Taiwan (*n*_AD_=15,495) (31), respectively, AD was associated with a decreased or similar risk of gestational diabetes. Likewise, the Danish study, using a matched control population, reported an elevated risk of gestational hypertensive disease among women with AD (5), whereas the Taiwanese study reported equal prevalences (31). Eclampsia (5) and preeclampsia were significantly associated with AD (31, 32), while one study found no association with preeclampsia (22). Threatened or completed miscarriage showed diverse results, as a significant association with AD was found in two studies from Taiwan and Japan (31, 33), whereas insignificant, conflicting findings were reported in another two studies (16, 34).

Considering preterm birth, using various definitions (see Table III), maternal AD was found to be protective in three studies (22, 35, 36), and indifferent in 2 studies (5, 34). Preterm premature rupture of membranes was associated with maternal AD in a Danish study (5), whereas a large Japanese questionnaire-based cohort study found no association (36). The risk of Staphylococcal neonatal septicemia was increased for women with AD in one study (5). Maternal AD was associated with small-for-gestational age at birth in a Japanese study (23), whereas 3 studies found no association to low birthweight (5, 22) or very low birthweight (21), respectively. Stillbirth was evaluated in three registry studies, of which AD was found protective in a Norwegian study evaluating 66,535 births by mothers with AD (22), while the other two studies (*n*_AD_=10,441 and *n*_AD_=3,261, respectively) found no association (5, 16).

## DISCUSSION

This systematic review provides a comprehensive overview of the existing literature on the impact of AD on different aspects of female reproduction. Overall, available studies indicate that while AD causes concerns regarding family planning, the majority of data do not demonstrate impaired fertility or adverse pregnancy or obstetric outcomes when compared with the general population.

A consistent finding across three out of four questionnaire-based studies was a high prevalence of concerns related to pregnancy among women with AD (4, 11, 24), and among men with AD (11). More than half of respondents reported anxiety about heritability (4, 11). Moreover, worries comprised treatment-related teratogenicity and potential disease worsening during pregnancy. In contrast, one of the Spanish studies found no impact of female or male AD on reproductive intentions or family planning decisions, regardless of disease severity (18). Whether it was the women or the man who had AD and the concern was on own AD or the partner's AD was not clear. Differences in healthcare systems, access to specialist counselling, cultural perceptions and disease burden may partly explain these discrepancies, although all 4 studies are recent and European. Importantly, all studies assessing concerns lacked reference groups, limiting conclusions regarding whether these worries are more prevalent among patients with AD than in the general population or other patient groups with chronic diseases. Likewise, women with rheumatic and skin diseases other than AD have reported high levels of concerns related to family planning (9). Nevertheless, the consistently high level of concern underscores a substantial unmet need for targeted reproductive counselling in this patient group.

Contrary to the hypothesis that women with AD conceive later in life, which may indirectly lead to impaired fertility, the available data do not unanimously support this. In fact, only a single Israeli nationwide cohort study reported a higher prevalence of infertility among adults with AD (19). In contrast, other recent large-scale registry studies found a lower risk of postponed pregnancy, lower likelihood of infertility treatments (28) and no difference considering assisted reproduction (17) in adults with AD compared with those without AD, respectively. However, the time to pregnancy was dichotomously evaluated with a cut-off of 12 months and thereby hindering full transparency of a potential delay. Pregnancy rates were not significantly correlated to AD although there was a tendency toward a lower rate in severe AD (16). Moreover, a large-scale registry study documented AD to be significantly associated with a higher fertility rate (30). Collectively, most of the studies suggest that AD is not linked with impaired fertility. Although the course of AD during pregnancy remains incompletely understood, speculations exist that immunological changes impact the pregnancy. During a normal pregnancy, the immune system shifts temporarily from a Type 1 Helper (Th1)- to a Th2-skewed immunity advantageous of implantation and early fetal tolerance (37). Theoretically, the Th2-skewed dominance in AD may therefore be immunologically favourable for maintenance of pregnancy and fertility rates.

The literature regarding changes in AD disease severity during pregnancy is inconsistent. Earlier retrospective studies reported deterioration in more than half of pregnancies (25, 26), while a more recent cross-sectional study found worsening in fewer than 20% (29). This apparent temporal shift might reflect improved disease management and greater awareness of pregnancy-safe therapies. Additionally, maternal stress was found to be associated with AD aggravation in late pregnancy in a prospective Swedish cohort, highlighting the bidirectional relationship between psychological burden and inflammatory skin disease (27). However, most of the included studies relied on self-reported disease activity, retrospective recall, and all were classified as low-to-moderate quality studies, limiting the robustness of these findings.

Notably, to our knowledge, no study directly evaluated maternal age at first childbirth, nor the number of children in women with AD, representing an important gap in the literature. However, childlessness at the age of 30 (17) or not having biological children at the age of 45 (20), respectively, were not associated with AD.

The adverse pregnancy and birth outcomes listed in this review are highly heterogeneous. The inverse association with gestational diabetes (5) is a new and surprising finding. In the Danish study sub-analysis indicated that a lower body mass index among AD compared with non-AD women might partly have driven the inverse association with gestational diabetes (5). Three out of four studies found maternal AD associated with preeclampsia (31, 32) and eclampsia (5), respectively. The association between preeclampsia and AD might be explained by natural killer cells and killer cell immunoglobulin-like receptor (*KIR*) genes, as both are suggested to play a role in the pathogenesis of AD and in preeclampsia (32, 38, 39).

Considering adverse birth outcomes, preterm birth was either inversely related with AD (22, 35, 36) or showed no association (5, 34). The authors of the studies speculated whether the potential protective effect of AD on nonpreterm pregnancy could be explained by the Th2 skewed profile in AD. In line with these findings, three studies documented no negative impact of AD on birth weight (5, 21, 22), although AD was associated with the small for gestational age in a single study (23). Moreover, stillbirth was less prevalent in AD (22) or found not related (5, 16). Collectively, the results indicate that AD is no disadvantage in these adverse birth outcomes or might even be protective. However, in line with the frequent skin colonization with *Staphylococcus aureus* in AD (40), one study identified an increased risk of Staphylococcal neonatal septicemia in women with AD (5). This is a serious complication, and awareness of these infections is important.

The strengths of this review include a systematic approach, inclusion of large population-based studies, and comprehensive coverage of multiple reproductive domains, which are collectively sparsely investigated. However, some limitations must be addressed. The included studies were predominantly observational or epidemiological and thereby precluding causal inference. The studies included were conducted differently regarding designs, population and outcomes, limiting comparisons and conclusions. A quality assessment of the studies was performed, although more homogeneous designs could have improved this process and enhanced comparability. Data on systemic treatment during pregnancy were out of the scope of this review as it was not the aim to evaluate potential risks using specific treatments.

Future research should prioritize prospective cohort studies integrating AD severity, treatment and reproductive intentions alongside objective fertility and pregnancy outcomes. Studies addressing maternal age at first childbirth, parity, and the impact of pregnancy counselling are needed to provide evidence-based guidance.

In summary, while women with AD frequently experience substantial concerns and psychological stress related to reproduction, current evidence supports that no adverse effects on fertility or pregnancy and birth outcomes should be expected. The discrepancy between patient-reported concerns and largely reassuring epidemiological data highlights the need for updated encouraging counselling, supporting women with AD throughout their reproductive years.

## Data Availability

The data that support the findings of this study are available from the corresponding author upon reasonable request.

## Supplementary material

Table SI.
