# Supplementary material for: Pregnancy in Women with Atopic Dermatitis: A Systematic Review of Concerns and Challenges
Source: Acta Derm Venereol. 2026 May 18;106:0608. doi: 10.2340/actadv.v106.adv-2026-0608 (PMC13184593; doi:10.2340/actadv.v106.adv-2026-0608)
Supplement: Table SI. [file ActaDv-106-0608-s0001.docx]

# SUPPLEMENTAL TABLE LEGENDS

## Table SI. Literature search scheme for PubMed and EMBASE.

|  | 1) Population: adults with atopic dermatitis  (combined with OR) | 2) Family planning, pregnancy, birth  (combined with OR) | 3) Challenges, concerns, adverse events  (combined with OR) |
| --- | --- | --- | --- |
| Text word | “atopic eczema”  “atopic dermatitis”  “Besnier’s prurigo” | Pregnan*  “family planning”  Reproduct*  “child bearing”  Fertility  Fecundity  Subfertility  Infertility  Conceiving  Birth | Concern*  Miscarriage  Infect*  “ectopic pregnancy”  “prolonged birth”  Problem*  Challenge*  Child*  Preeclampsia  Complication*  “adverse event*”  “gestational diabetes”  Preterm  “birth weight”  Treatment  “disease worsening” |
| MeSH (PubMed) or keywords (EMBASE) | “Dermatitis, Atopic” | Fertility  Infertility  Parturition | “abortion, spontaneous”  “pre eclampsia”  “diabetes, gestational”  “birth weight”  therapeutics |
| Search strategy: 1 AND 2 AND 3 limited to human studies in English language  *search mark that counts plural and other last letters as well. | | | |
